# Plantar Vein Thrombosis: An Unusual Cause of Plantar Pain

**DOI:** 10.5334/jbr-btr.874

**Published:** 2015-12-30

**Authors:** M. Vansevenant, F. M. Vanhoenacker

**Affiliations:** 1 Department of Radiology, AZ Sint-Maarten, Mechelen-Duffel, Duffel, Belgium; 2 Department of Radiology, Ghent University Hospital (UZ Gent), Ghent, Belgium; 3 Department of Radiology, Antwerp University Hospital (UZA), Edegem, Belgium

**Keywords:** Plantar vein thrombosis, ultrasound, MRI

## Abstract

We present a case of an 80-year-old man with progressive pain for 5 days at the medial and plantar aspect of the left heel. Wearing shoes aggravated the pain. Ultrasound and magnetic resonance imaging (MRI) revealed thrombosis of the medial plantar veins. Plantar vein thrombosis is a rare condition. The clinical symptoms are non-specific and can be confused with plantar fasciitis. It has been associated with hypercoagulable conditions, foot trauma and recent surgery. The imaging modality of choice is ultrasound. MRI may add to the diagnosis in unclear cases.

## Introduction

Plantar heel pain is a very common complaint, accounting for up to 11% to 15% of foot and ankle disorders. Most of these patients with plantar heel pain suffer from plantar fasciitis [1]. The purpose of this manuscript is to report a rare cause of plantar heel pain resulting from plantar vein thrombosis.

## Case

An 80-year-old man with progressive pain lasting for 5 days and with focal swelling of the left foot was referred to the radiology department. Wearing shoes aggravated the pain. Inspection of his footwear showed a bump at the inner sole, corresponding with the location of the clinical abnormality at the heel of the patient. Clinically, there was suspicion of plantar fasciitis.

**Figure 1:**
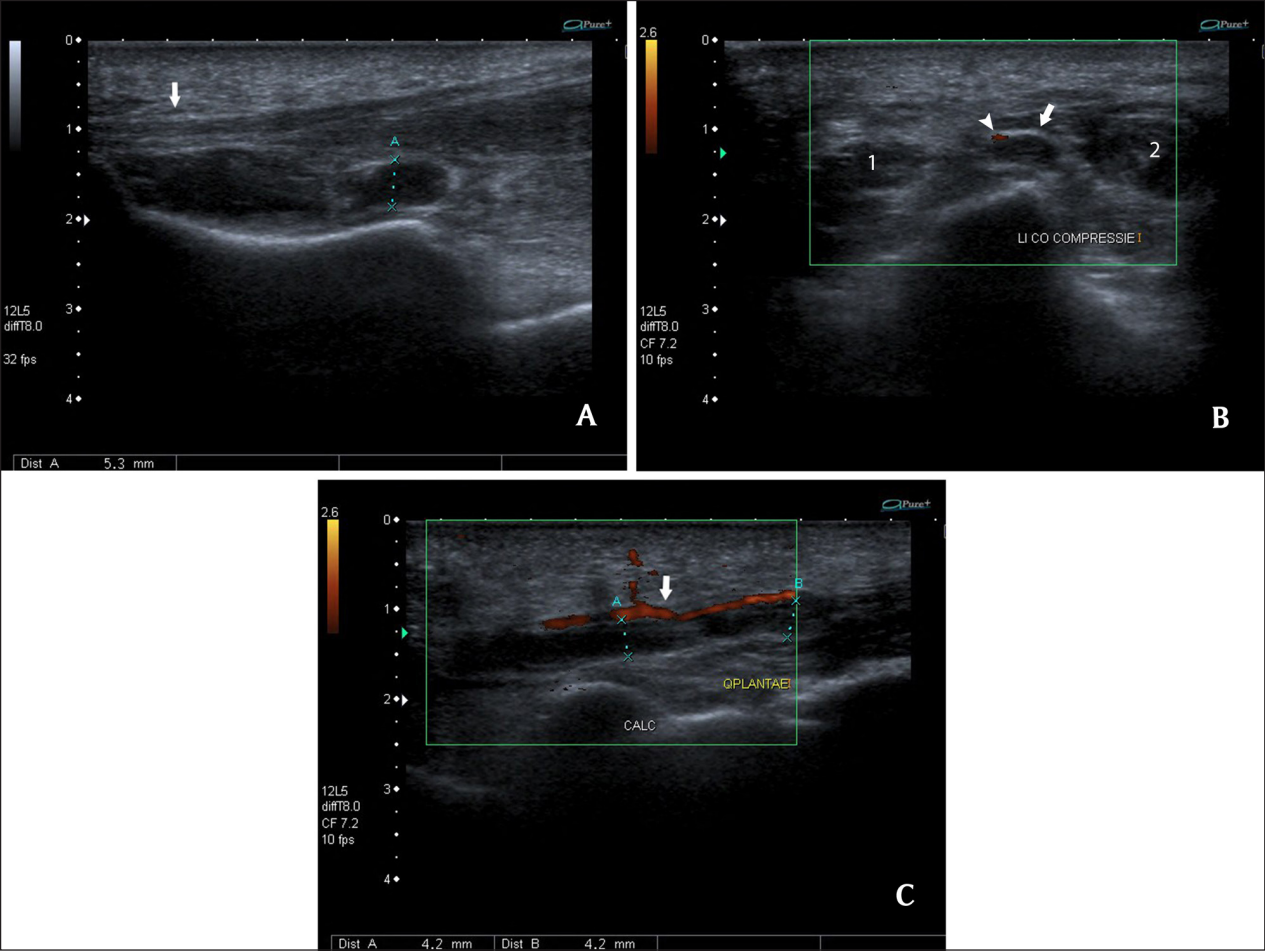
Ultrasound of the left foot. Sagittal view (A). There is a normal thickness of the plantar fascia between 2 and 4 mm (arrow). Tubular, hypoechoic structures are seen near the plantar fascia. Coronal view (B) with compression and color Doppler imaging. These structures are not compressible, and there is no blood flow. The thrombus (arrow) is located deeply and medially to the plantar artery (arrowhead) and between the musculus (m.) flexor digitorum brevis (1) and m. abductor hallucis (2). Sagittal view with color Doppler imaging (C). The thrombi (B-C) are located deeply beneath the medial plantar artery (arrow).

**Figure 2:**
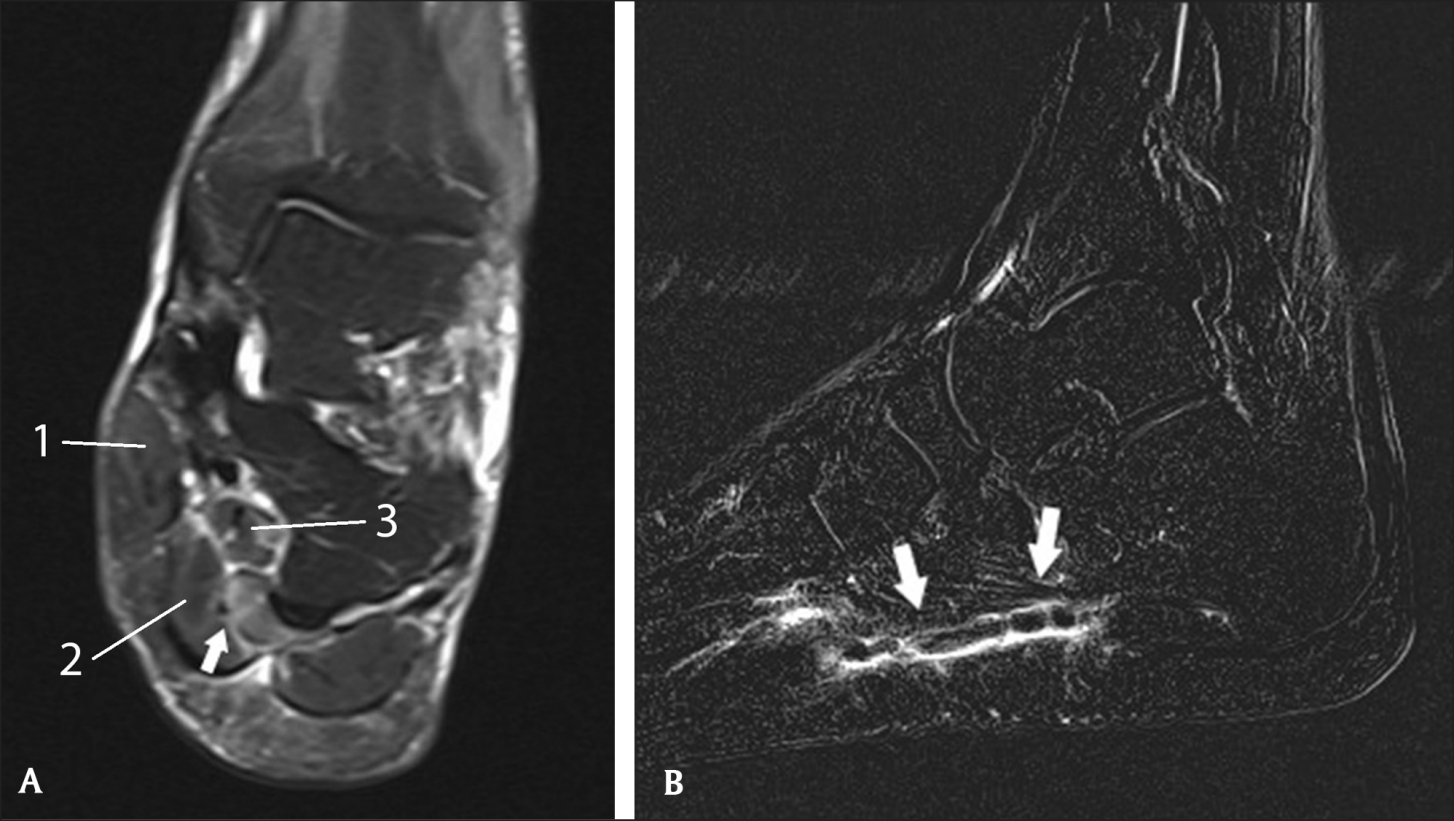
MRI of the left foot. Coronal reformatted T2-WI FS (A) showing subtle intramuscular edema (arrow) adjacent to the medial plantar veins. The thrombosed vein itself is not visible. Medially from the medial plantar artery and veins is the m. abductor hallucis (1); laterally the m. flexor digitorum brevis (2); and deeply beneath is the m. quadratus plantae (3). Sagittal reformatted T1-weighted subtraction image after gadolinium (B). There is a filling defect in the medial plantar veins (arrows) with a thin sleeve of hyperintense contrast around the thrombus.

Plain films showed the absence of inferior calcaneal spur formation. Ultrasound revealed a normal plantar fascia. Medially from the plantar fasciitis, adjacent to the course of the medial plantar artery, hypoechoic tubular structures were seen, which were not compressible. There was no intralesional flow on color Doppler imaging (Figure 1). Comparison with the right foot showed normal compressible veins. Subsequent MRI confirmed thrombosis of the medial plantar veins (Figure 2).

The symptoms disappeared after conservative therapy with change of footwear and non-steroidal anti-inflammatory drugs. Follow-up ultrasound 2 months later revealed no residual thrombus in the plantar veins.

## Discussion

Plantar vein thrombosis has been considered a very rare cause of plantar foot pain because the condition has been reported rarely as cause of plantar pain [2,3,4,5]. However, the frequency of the condition might be underestimated. Most often, plantar heel pain is attributed to mechanical problems, resulting in plantar fasciitis [1]. The symptoms of plantar vein thrombosis are non-specific and include plantar foot pain, local swelling [2,3,4,5] and pain increasing during walking [4].

The deep plantar veins are divided in the lateral and medial plantar veins, accompanying the lateral and medial plantar artery. These two veins initiate at the first intermetatarsal space. They are connected at this first intermetatarsal space with the superficial venous system. At the medial malleolus, they drain to the posterior tibial vein [2,3,4,5]. The lateral plantar veins are more affected by thrombosis than the medial plantar veins are [4,5].

Virchow proposed a triad to describe the potential causes of venous thrombosis. This triad has been modified to the following factors: blood coagulability, changes in the vessel wall (inflammation and friction) and stasis. Various diseases, such as genetic disorders in the making of coagulation factors or neoplastic syndromes, as well as the intake of oral contraceptives, cause increased coagulability of blood [6].

In the literature, cases of plantar vein thrombosis have been reported in patients with clotting diseases and paraneoplastic syndromes, after trauma or in postoperative states (immobilization and after saphenectomy) [2,3,4,5]. To the best of our knowledge, there are no known cases of plantar vein thrombosis resulting from wearing inappropriate footwear. In our case, a possible explanation for the plantar vein thrombosis is venous stasis of blood and local trauma caused by the hump on the inner sole of the shoe of the patient.

**Figure 3:**
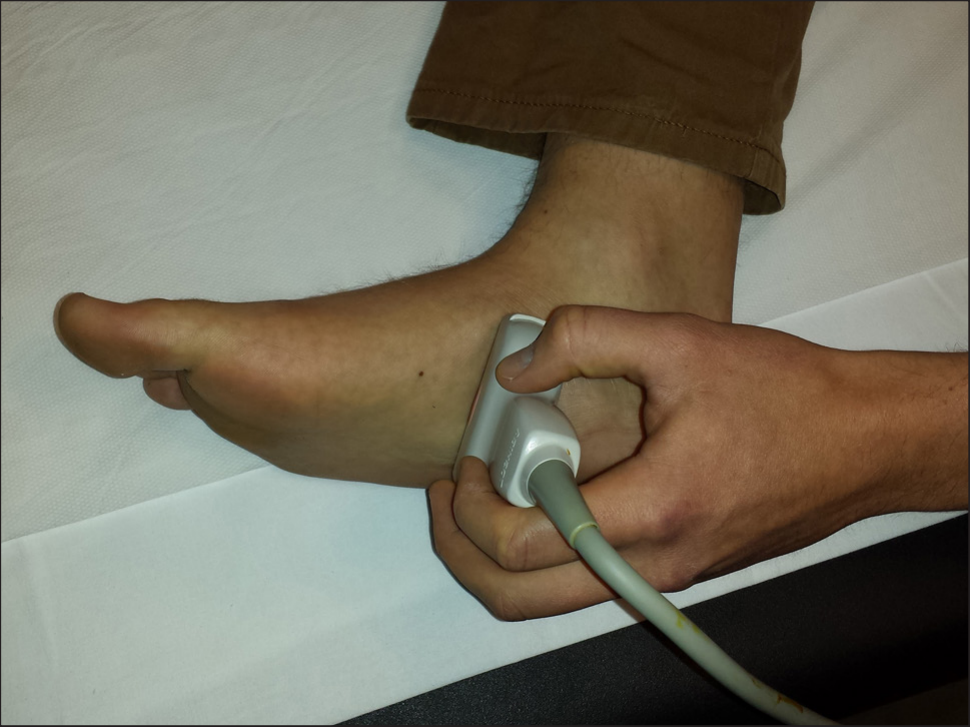
Position of the ultrasound probe for visualization of the medial plantar veins in a coronal plane. To visualize the medial plantar veins in a sagittal plane, the probe is turned 90° in a longitudinal direction on the foot.

**Figure 4:**
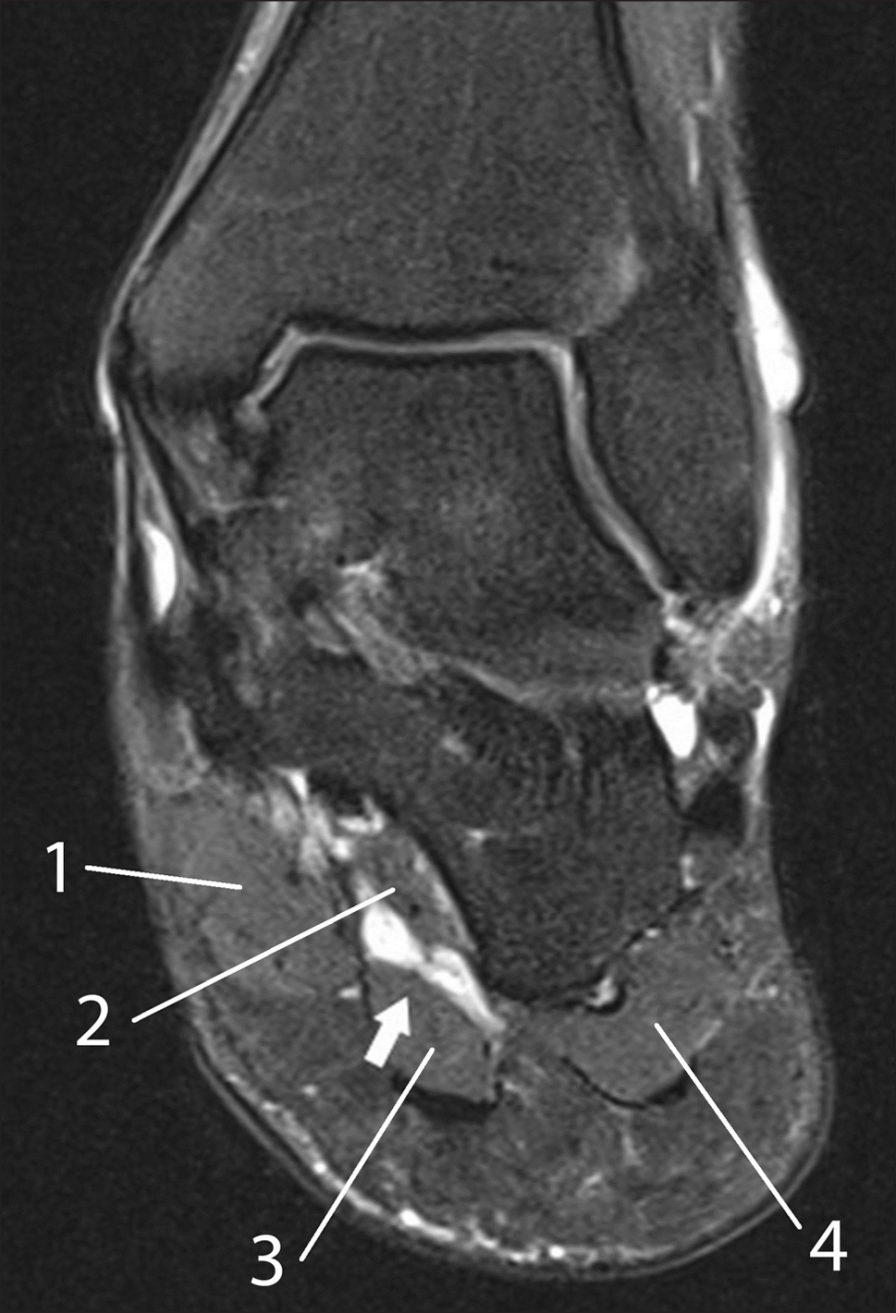
MRI of the left foot in a normal patient for comparison. Coronal reformatted T2-WI showing clearly the hyperintense medial plantar veins (arrow) in normal conditions. The veins are surrounded by the m. abductor hallucis (1) medially; the m. quadratus plantae (2) deeply from the veins; and the m. flexor digitorum brevis (3) laterally. The muscle belly of the m. abductor digiti minimi (4) is located at the lateral plantar aspect of the foot.

The preferred imaging modality for the diagnosis of plantar vein thrombosis is ultrasound (Figure 3). The presence of noncompressible veins, adjacent to the medial or lateral plantar artery (which can be used as an anatomical landmark), is the hallmark of the disease. On color Doppler ultrasound, there is absence of venous flow [2,3,4,5]. MRI is usually not required, but it can be helpful in obese patients or patients with horny plantar skin. In case of thrombosis, there is a filling defect on T1-weighted images (WI) after administration of gadolinium contrast, and absence of hyperintense veins on T2-WI. In normal conditions, the plantar veins are hyperintense on T2-WI, particularly if fat suppression is applied (Figure 4). In addition, subtle perivascular edema in the muscles adjacent to the plantar veins can be seen [2,4].

## Conclusion

Although relatively rare, plantar vein thrombosis should be considered in the differential diagnostic list of plantar pain, particularly in the appropriate clinical setting. Systematic and meticulous inspection of the plantar veins on ultrasound and MRI is warranted to recognize this likely underestimated disease.

## Competing Interests

The authors declare that they have no competing interests.
